# Single-cell RNA sequencing of neural stem cells derived from human trisomic iPSCs reveals the abnormalities during neural differentiation of Down syndrome

**DOI:** 10.3389/fnmol.2023.1137123

**Published:** 2023-06-15

**Authors:** Jia-jun Qiu, Yan-na Liu, Hao Wei, Fanyi Zeng, Jing-bin Yan

**Affiliations:** ^1^Shanghai Children’s Hospital, Shanghai Institute of Medical Genetics, Shanghai Jiao Tong University School of Medicine, Shanghai, China; ^2^Department of Hiso-Embryology, Genetics and Developmental Biology, Shanghai Jiao Tong University School of Medicine, Shanghai, China; ^3^NHC Key Laboratory of Medical Embryogenesis and Developmental Molecular Biology and Shanghai Key Laboratory of Embryo and Reproduction Engineering, Shanghai, China

**Keywords:** single-cell sequencing, Down’s syndrome, neural stem cell, induced pluripotent stem cell, neural differentiation

## Abstract

**Introduction:**

Down syndrome (DS) is the most common genetic condition that causes intellectual disability in humans. The molecular mechanisms behind the DS phenotype remain unclear. Therefore, in this study, we present new findings on its molecular mechanisms through single-cell RNA sequencing.

**Methods:**

Induced pluripotent stem cells (iPSCs) from the patients with DS and the normal control (NC) patients were differentiated into iPSCs-derived neural stem cells (NSCs). Single-cell RNA sequencing was performed to achieve a comprehensive single-cell level differentiation roadmap for DS-iPSCs. Biological experiments were also performed to validate the findings.

**Results and Discussion:**

The results demonstrated that iPSCs can differentiate into NSCs in both DS and NC samples. Furthermore, 19,422 cells were obtained from iPSC samples (8,500 cells for DS and 10,922 cells for the NC) and 16,506 cells from NSC samples (7,182 cells for DS and 9,324 cells for the NC), which had differentiated from the iPSCs. A cluster of DS-iPSCs, named DS-iPSCs-not differentiated (DSi-PSCs-ND), which had abnormal expression patterns compared with NC-iPSCs, were demonstrated to be unable to differentiate into DS-NSCs. Further analysis of the differentially expressed genes revealed that inhibitor of differentiation family (ID family) members, which exhibited abnormal expression patterns throughout the differentiation process from DS-iPSCs to DS-NSCs, may potentially have contributed to the neural differentiation of DS-iPSCs. Moreover, abnormal differentiation fate was observed in DS-NSCs, which resulted in the increased differentiation of glial cells, such as astrocytes, but decreased differentiation into neuronal cells. Furthermore, functional analysis demonstrated that DS-NSCs and DS-NPCs had disorders in axon and visual system development. The present study provided a new insight into the pathogenesis of DS.

## Introduction

Down syndrome (DS) is caused by trisomy of chromosome 21 (*Homo sapiens* 21) and is the most common genetic condition and the leading genetic cause of intellectual disability in humans. DS has an incidence rate of approximately 1/700–1,000 live births worldwide (Qiu et al., 2017). The DS phenotype is always characterized by neurodevelopmental anomalies and early neurodegenerative processes (Hibaoui et al., 2014). Several brain abnormalities have been observed in patients with DS, including reduced gross brain weight, a lower number and depth of cerebral sulci, enlarged ventricles, and hypoplasia of several brain structures, including the brainstem, cerebellum, and frontal and temporal lobes (Annus et al., 2017).

However, the underlying molecular mechanisms of the DS phenotype remain unclear due to a lack of suitable models that contain the full trisomy of chromosome 21 (Ts21) in a human genome. Transgenic mice models are also not perfect and it is hard to obtain human tissues at the appropriate neurodevelopmental stages (Brigida and Siniscalco, 2016). To overcome these limitations, human induced (i) pluripotent stem cells (PSCs) derived from patients with DS are used in research concerning the molecular and biological consequences of Ts21 (Weick et al., 2013). iPSC technology enables the modeling of complex diseases, including DS, in the context of human cell biology (Mizuno et al., 2018), which can generate disorder-specific human cells to simulate the phenotype of human disease (Weick et al., 2016). The neural stem cells (NSCs) derived from DS-iPSCs provide a robust platform to study the neurodevelopment of DS and they have the potential to generate neurons, oligodendrocytes and astrocytes (Lam et al., 2019).

Genome-wide perturbations of gene expression have previously been reported in DS-iPSCs and DS-iPSC-derived neural cells, based on bulk transcriptomics (Weick et al., 2013; Qiu et al., 2017; Ponroy Bally et al., 2020). However, as the basic unit of organisms, single cells have cell-to-cell diversity, heterogeneity, and dynamics. Previous studies have also demonstrated that human iPSC-derived NSCs contain different subpopulations with varying potentials for multipotency of differentiation into neurons, oligodendrocytes, and astrocytes (Lam et al., 2019). Therefore, although the differentiation of iPSCs can be used to mimic disease phenotype at different neurodevelopmental stages, bulk transcriptomics studies at the cell-population level, including microarrays and bulk RNA sequencing, will lose the omics information from each cell. However, with the arrival of single-cell RNA sequencing (scRNA-seq) technology, it is possible to analyze individual cells from heterogeneous cell subpopulations at a single-cell resolution.

In the present study, iPSCs from a patient with DS were induced and differentiated into NSCs. scRNA-seq was performed to analyze the subpopulations of NSCs and iPSCs and the high-throughput transcriptional composition and mapped gene expression profiles were obtained. The results of the present study may have provided new insight into the mechanism of DS in neurodevelopment.

## Materials and methods

### Human iPSC culture

Down syndrome-induced pluripotent stem cell and normal control (NC)-iPSC lines were purchased from the American Type Culture Collection (cat. no. ACS-1011, healthy newborn male; and cat. no. ACS-1003, DS newborn male). To confirm the results, another DS-iPSC and NC-iPSC lines (named DS-iPSCs-2 and NC-iPSCs-2), which were prepared by nuwacell Biotechnologies Co., Ltd. (Hefei, China), were used. Both cell lines were generated from dermal fibroblast cells. Cells were cultured on Geltrex-coated dishes (Gibco; Thermo Fisher Scientific, Inc.) in a 5% CO2 atmosphere at 37°C and maintained with daily media changes using serum-free media StemFlex (Gibco; Thermo Fisher Scientific, Inc.). Cells were passaged using Gentle Cell Dissociation Reagent (Stemcell Technologies, Inc.) once 70–80% confluency was reached.

### Differentiation from iPSCs to NSCs

Once cells reached 20% confluency after splitting, the culture medium was switched to complete PSC Neural Induction Medium containing Neurobasal Medium and Neural Induction Supplement (Gibco; Thermo Fisher Scientific, Inc.). The neural induction medium was changed on days 2 and 6 of neural induction. On day 7 of neural induction, primitive NSCs were dissociated and plated on Geltrex-coated dishes at a density of 0.5–1 × 10^5^ cells/cm^2^ Neural Expansion Medium containing Neurobasal® Medium, Advanced™ DMEM/F-12 (Gibco; Thermo Fisher Scientific, Inc.), and Neural Induction Supplement. Subsequently, 5 μM rho-kinase inhibitor Y27632 (Sigma-Aldrich; Merck KGaA) was added to the neural expansion medium at the time of NSC plating. The neural expansion medium was changed every other day until NSCs reached confluence on day 6 of plating.

### Characterization of iPSCs and NSCs with immunofluorescence and flow cytometry analysis

Immunofluorescence was used to identify the different cell types of iPSCs and NSCs. Cells were plated on Matrigel-coated slides and were fixed with 4% paraformaldehyde at room temperature for 30 min. After rinsing with PBS, cells were permeabilized and blocked using a blocking buffer containing 0.2% Triton X-100 (Sigma-Aldrich; Merch KGaA) and QuickBlock™ Blocking Buffer for Immunol Staining (Beyotime Biotechnology, Inc.) at room temperature for 30 min. Following incubation with the primary antibodies at 4°C overnight in the blocking buffer and three rinses in PBS, Alexa Fluor 488 (cat.no. A0423; Beyotime) and/or Cy3-conjugated secondary antibodies (cat.no. A0521; Beyotime) were used to visualize stained cells. Cell nuclei were counterstained with DAPI. Fluorescent images were captured using an inverted fluorescence microscope.

To analyze the cell types in NSCs, flow cytometry analysis (FACS) was performed. Following incubation with the primary antibodies and secondary antibodies, the cells were analyzed on the CytoFLEX cytometers (Beckman Coulter Life Science, Inc.) and analyzed by FlowJo software (Tree Star, United States).

The following primary antibodies were used: The pluripotent marker TRA-1-60 (cat. no. ab16288; Abcam); NSC markers, including paired box 6 (PAX6; 1:350; cat. no. ab195045; Abcam), Nestin (1:250; cat. no. MAB1259-SP; R&D Systems, Inc.), and SOX1 (10 μg/mL; cat. no. AF3369; R&D Systems, Inc.).

### Single-cell RNA sequencing and data analysis

Single-cell RNA sequencing was performed by Novogene Co., Ltd. using a 10x Genomics Chromium system and the Illumina Hiseq PE150 protocol. Cell Ranger Single Cell Software Suite 3.0 (10x Genomics) was used to process the sequencing data into transcript count tables. The “Cellranger mkfastq” pipeline demultiplexed raw base call files generated by Illumina sequencers into sample-specific FASTQ files. Subsequently, “Cellranger count” used FASTQ files as input and aligned the reads onto the hg38 human reference using Spliced Transcripts Alignment to a Reference. After filtering, barcode counting and the unique molecular identifiers (UMI) counting process, the “cellranger aggr” pipeline was used to combine data from multiple samples into an experiment-wide feature-barcode matrix for downstream analysis.

Seurat 4 (version 4.1.0) standard analysis pipeline was applied to perform the cell clustering (Hao et al., 2021). The “IntegrateData” function was used to merge DS and normal samples based on the anchors identified via the “FindIntegrationAnchors” function. Principal component analysis was performed based on the scaled data and the top 20 principals were used for dimension reduction with the uniform manifold approximation and projection method. Furthermore, the cell clusters were identified using the “FindClusters” function. The Seurat “FindAllMarkers” function was used to identify conserved markers that were upregulated in each cluster vs. all other cells (adjusted *p* < 0.05). The CellCycleScoring function was used to remove the cell cycle effect. Moreover, cell clusters were annotated using SCSA with the CellMarker database based on the marker genes (Cao et al., 2020). Pseudo-time trajectory analysis was performed using the Monocle2 package (Trapnell et al., 2014).

Differential expression analysis was performed using the Seurat “FindMarkers” function (adjusted *p* < 0.01; |log(Fold Change)| > 0.5). Heatmap plots were drawn using the DittoSeq “dittoHeatmap” function. Gene ontology (GO) enrichment analysis was performed using the clusterProfiler package (Wu et al., 2021). Moreover, *p* < 0.001 indicated the extremely small-adjusted *p* value.

Then, we validated part of our analysis based on an independent dataset (Palmer et al., 2021). We plotted the gene expression among the cell sub-populations, which were identified by authors in the previous study.

### RNA extraction and reverse transcription-quantitative PCR

Total RNA from each sample was extracted using TRIzol^®^ reagent (Gibco; Thermo Fisher Scientific, Inc.), and complementary DNA was synthesized using random primers and the PrimeScript RT Reagent Kit (Takara Bio, Inc.). RT-qPCR was performed using the One-Step TB Green PrimeScript RT-PCR Kit (Takara Bio, Inc.). Primers for the RT-qPCR experiment were designed using Primer 5.0 software. The GAPDH gene was used to standardize the expression levels. The sequences of the primers were as follows: GAPDH forward (F), 5′-CGGAGTCAACGGATTTGGTCGTAT-3′ and reverse (R), 5′-AGCCTTCTCCATGGTGGTGAAGAC-3′; inhibitor of differentiation (ID)1 F, 5′-CTGGACGAGCAGCAGGTAAA-3′ and R, 5′-AGGAACGCATGCCGCC-3′; ID2 F, 5′-TGAAAGCCTTCAGTCCCGTG-3′ and R, 5′-TGGTGATGCAGGCTGACAAT-3′; and ID3 F, 5′-TGGAAATCCTACAGCGCGTC-3′ and R, 5’-CTGCGTTCTGGAGGTGTCAG-3′. All reactions were replicated three times for each sample. The qPCR results were analyzed using the 2^-ΔΔCq^ method. The paired Student’s *t*-test was performed on the RT-qPCR data and *p* < 0.05 was considered to indicate a statistically significant difference.

## Results

### iPSCs can differentiate into NSCs in both DS and NC samples

**Figure 1:**
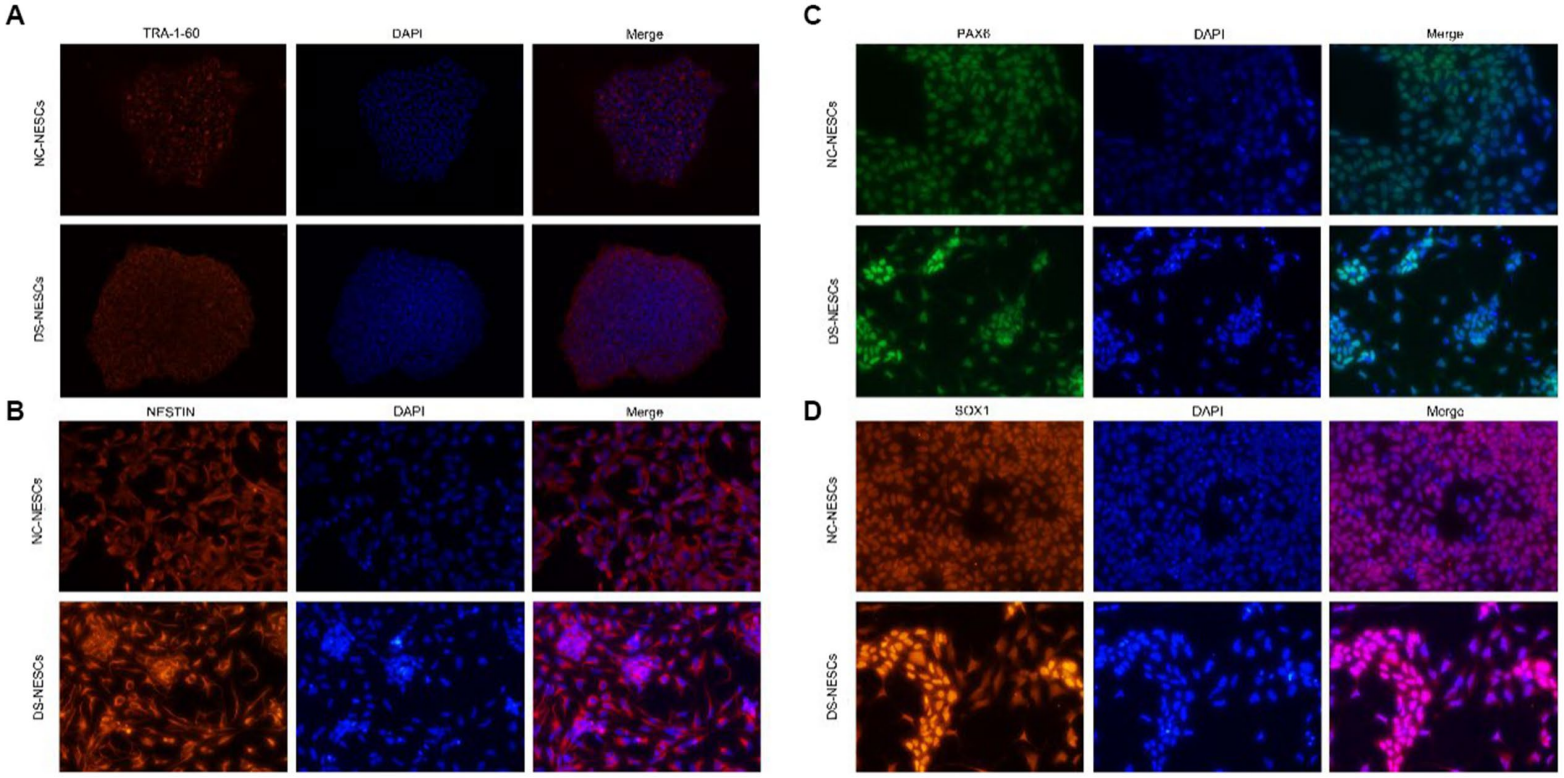
Immunofluorescence detection of stemness markers in iPSCs and differentiation markers in NSCs. **(A)** Expression of TRA-1-60 was assessed via immunofluorescence and confocal microscopy in both DS-iPSC and NC-iPSC clones. Representative images demonstrated that both cell types were positive for the anti-TRA-1-60 antibody (red). Nuclei were also stained with DAPI (blue). **(B–D)** Protein expression levels of Nestin, PAX6, and SOX1 were assessed using immunofluorescence and confocal microscopy in both NC-NSCs and DS-NSCs. Representative images demonstrate that both cell types were positive when stained with the Nestin antibody (red), PAX6 antibody (green), and SOX1 antibody (red). Nuclei were also stained with DAPI (blue). iPSC, induced pluripotent stem cell; NSC, neural stem cell; DS, Down syndrome; NC, normal control; and PAX6, paired box 6.

It was first to determine whether NC-iPSCs and DS-iPSCs had classical morphological features. Phase-contract microscopy images indicated that iPSC clones had the typical morphology of iPSCs with distinct colony boundaries. Moreover, all the iPSCs clones expressed significant levels of the pluripotency marker TRA-1-60 (Figure 1A).

After 10 days of induction, iPSCs were differentiated into NSCs. Phase-contract microscopy demonstrated that the morphology of DS-NSCs was different from that of NC-NSCs. NC-NSCs that differentiated from NC-iPSCs were evenly distributed. However, DS-NSCs were unevenly distributed and were aggregated. Both NC-NSCs and DS-NSCs expressed the markers of neural differentiation, NESTIN, PAX6, and SOX1 (Figures 1B–D).

### scRNA-seq in iPSCs and NSCs

Following sequencing and quality control analysis, 1,135,270,267 sequence reads for 19,422 cells from two iPSCs samples (8,500 cells for DS and 10,922 cells for the NC sample) and 964,233,383 sequence reads for 16,506 cells from two NSC samples (7,182 cells for DS and 9,324 cells for the NC sample) were obtained, which were differentiated from the iPSCs. iPSCs were sequenced with an average depth of 58,453 reads per cell (RPC) and NSCs with 58,417 RPC. Subsequently, the low-quality cells based on the number of genes and UMIs and the percentage of expressed mitochondrial genes were removed (Supplementary Table S1). Finally, for downstream analysis, there were 4,188 and 5,427 iPSCs for DS and NC samples, respectively. Moreover, for NSCs, there were 5,398 and 5,088 cells for DS and NC samples, respectively.

### DS-NSCs have an abnormal differentiation fate compared with NC-NSCs

**Figure 2:**
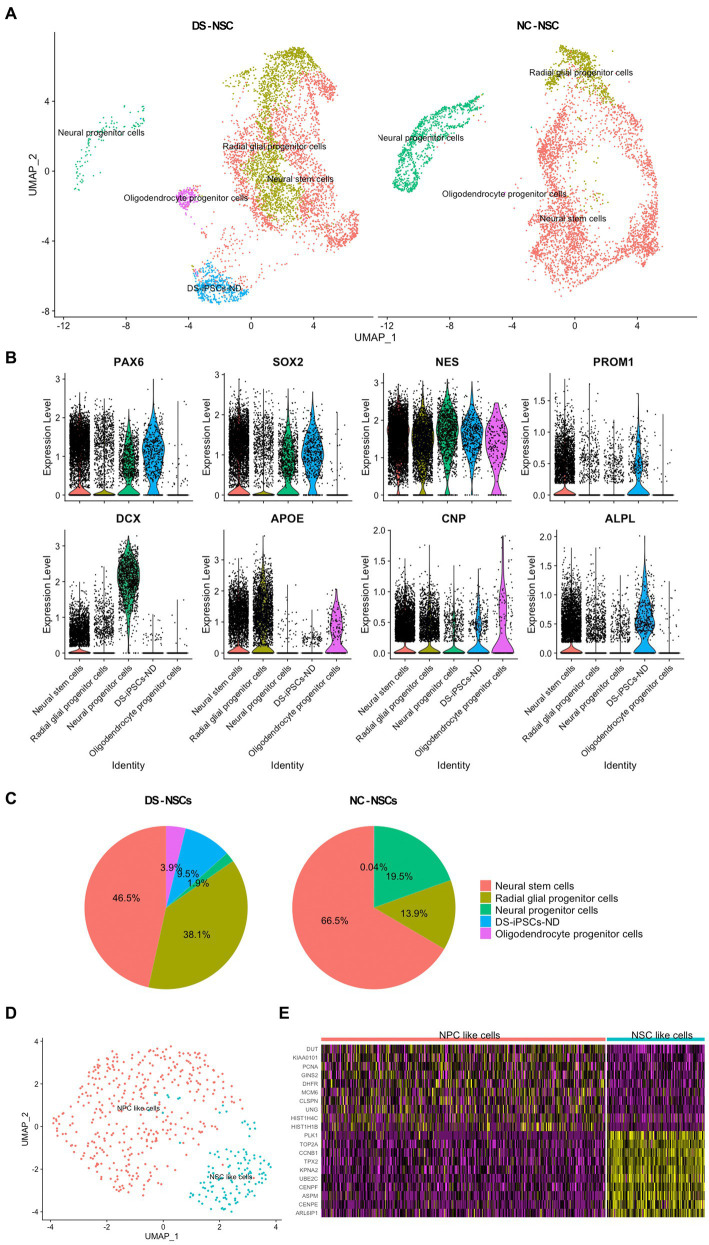
Identification of the subpopulations in NSCs and the abnormal differentiation fate of DS-NSCs. **(A)** Uniform manifold approximation and projection plots present the various subpopulations in NC-NSCs and DS-NSCs, including NSCs, NPCs, radial glial progenitor cells, oligodendrocyte progenitor cells, and DS-iPSCs-ND. Among them, DS-iPSCs-ND only existed in the DS-NSC group. **(B)** Expression levels of marker genes among subpopulations. **(C)** Abnormal distribution of the subpopulations in DS-NSCs compared with NC-NSCs. **(D)** Two subpopulations in DS-iPSCs-ND cells: (i) NSC-like cells; and (ii) NPC-like cells. **(E)** Expression levels of marker genes in two subpopulations of DS-iPSCs-ND. NSC, neural stem cell; NPC, neural progenitor cells; NC, normal control; DS, down syndrome; iPSC, induced pluripotent stem cell; and ND, not differentiated.

After merging the DS-iPSCs and NC-iPSCs-derived NSC data, cell subpopulations were identified based on the marker genes expressed in each cell cluster (Figure 2A).

Down syndrome- and NC-iPSC-derived NSCs were demonstrated to share most subpopulations. They all contained both neurogenic and gliogenic progenitors, which was consistent with previous studies as NSCs were highly expandable with the potential for multipotency of differentiation into neurons, oligodendrocytes, and astrocytes. Known pan-differentiation markers of neural stem cells (*PAX6, SOX2*, and *NESTIN*) were expressed in both neurogenic and gliogenic progenitors (Figure 2B). Moreover, each subpopulation expressed its marker gene. NSCs expressed *CD133* along with the three pan-differentiation markers. There was also a high expression level of doublecortin (*DCX*) in neural progenitor cells (NPCs). Radial glial progenitor cells (RGPCs) and oligodendrocyte progenitor cells also expressed their marker genes, apolipoprotein E and 2′,3′-cyclic nucleotide 3′phosphodiesterase (*CNP*).

Subsequently, an abnormal distribution of NSCs for DS samples was observed. NC-NSCs consisted of 66.5% NSCs and 19.5% NPCs (Figure 2C). However, the fractions in DS-NSCs were only 46.5% NSCs and 1.9% NPCs. In contrast to the under-representation of NPCs, which are the source of differentiation potential in neurons, DS-NSCs had a large fraction of RGPCs (38.1%). However, there were only 13.9% RGPCs in the NC-NSCs. These results indicated that DS-NSCs exhibited increased differentiation into glial cells, such as astrocytes, but decreased differentiation into neuronal cells.

Furthermore, the results also demonstrated that 9.5% of DS-iPSCs were not able to differentiate into NSCs, which were named DS-iPSCs-not differentiated (ND). DS-iPSCs-ND expressed differentiation markers, such as *PAX6* and *NESTIN*. However, they also expressed numerous markers of iPSCs similar to DS-iPSCs and NC-iPSCs, including *LIN28*, alkaline phosphatase biomineralization-associated, *CD9*, *CD24*, and *CD133*, but they had lost two iPSCs markers, *NANOG* and *OCT3/4* (Supplementary Figure S1). The DS-iPSCs-ND cells could be further divided into two sub-clusters: (i) NSC-like cells, whose expression profile was close to that of NSCs; and (ii) NPC-like cells, which were similar to NPCs (Figures 2D,E). These results suggested that DS-iPSCs-ND were DS-iPSCs that had been stopped during the process of differentiation into NSCs.

Subsequently, further comprehensive research was performed to investigate the properties of DS-NSCs and to explore the four major cell subpopulations: DS-iPSCs-ND, NSCs, DS-RGPCs, and NPCs.

### Differentiation process of DS-iPSCs-ND into NSCs was repressed due to multiple reasons

It was demonstrated that a subset of DS-iPSCs were unable to differentiate into NSCs and this was therefore further investigated. The DS-iPSC and DS-NSC datasets were merged and trajectory analysis was performed.

**Figure 3:**
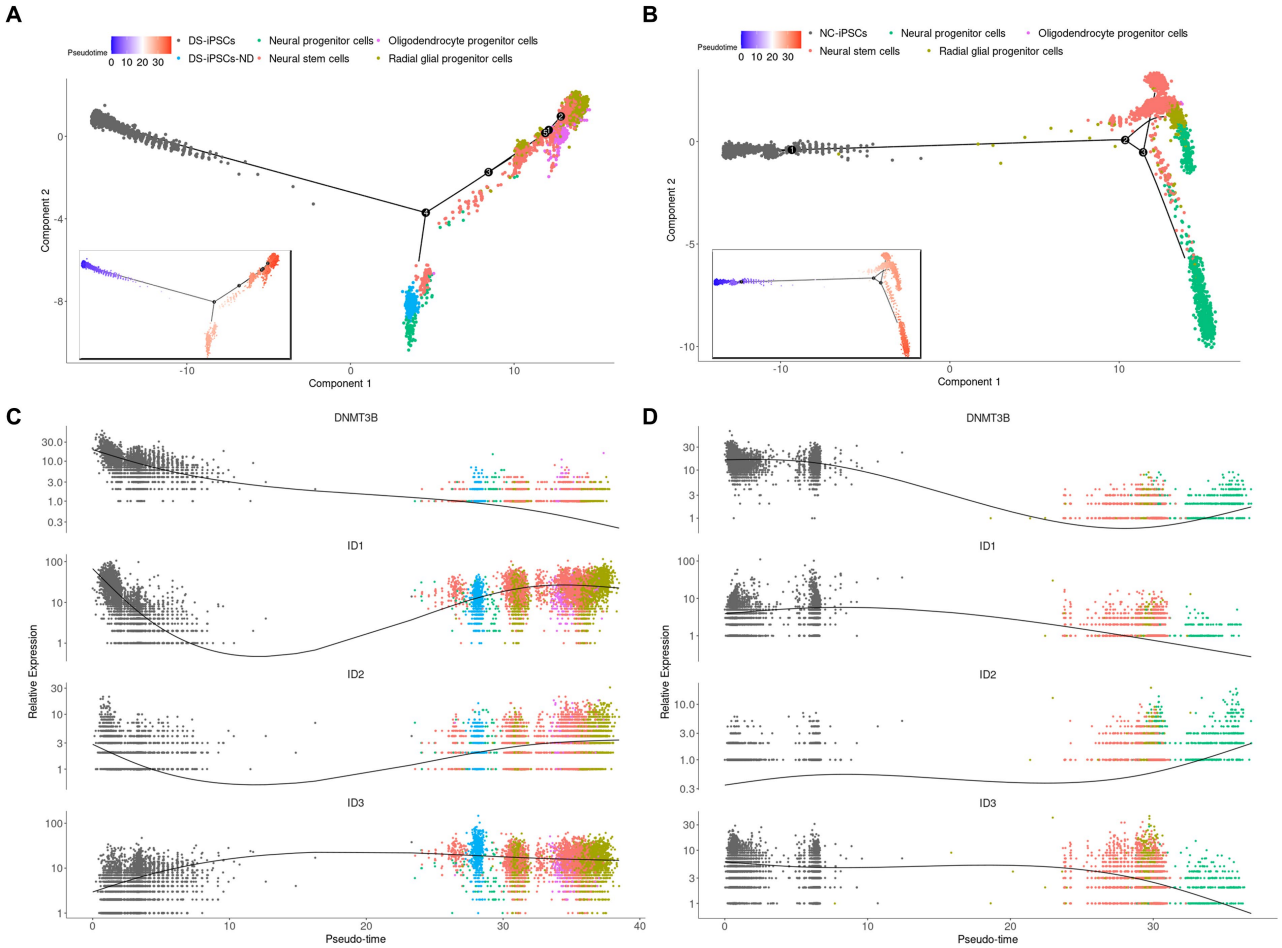
Pseudo-time trajectory analysis for DS-NSCs and NC-NSCs. Pseudo-time trajectory analysis for **(A)** DS-NSCs and **(B)** NC-NSCs. Pseudo-time color changes from blue (early) to red (later). Gene expression profiles throughout the pseudo-time trajectory analysis for *DNMT3B*, *ID1*, *ID2*, and *ID3* in **(C)** DS-NSCs and **(D)** NC-NSCs. DS, Down syndrome; NSC, neural stem cell; NC, normal control; DNMT3B, DNA methyltransferase 3β; and ID, inhibitor of differentiation.

DS-iPSCs-ND were clearly mapped on DS-iPSCs and separated from the other cells, which indicated DS-iPSCs-ND had a similar status and expression profile compared with DS-iPSCs (Supplementary Figure S2). Moreover, the trajectory analysis was performed on both DS (Figures 3A,C) and NC (Figures 3B,D) cells. To make a clear demonstration, we also plotted the expression of genes among different discrete time states, which were identified by Monocle (Supplementary Figure S3). The results demonstrated that DS-iPSCs-ND cells were located in the middle between the DS-iPSCs and differentiated DS-NSCs on the pseudo-timeline, which confirmed the hypothesis that a subset of DS-iPSCs were unable to differentiate into NSCs and were repressed in the middle of the differentiation process.

To further confirm whether DS-iPSCs-ND was common in cell subpopulations of DS-NSCs. The cell markers were analyzed in two independent DS-NSCs by FACS. The results showed that the pluripotency marker TRA-1-60 was more commonly expressed (Supplementary Figure S4A). However, the proportion of cells expressing neural markers (PAX6) decreased (Supplementary Figure S4B) in two independent DS-NSCs.

**Figure 4:**
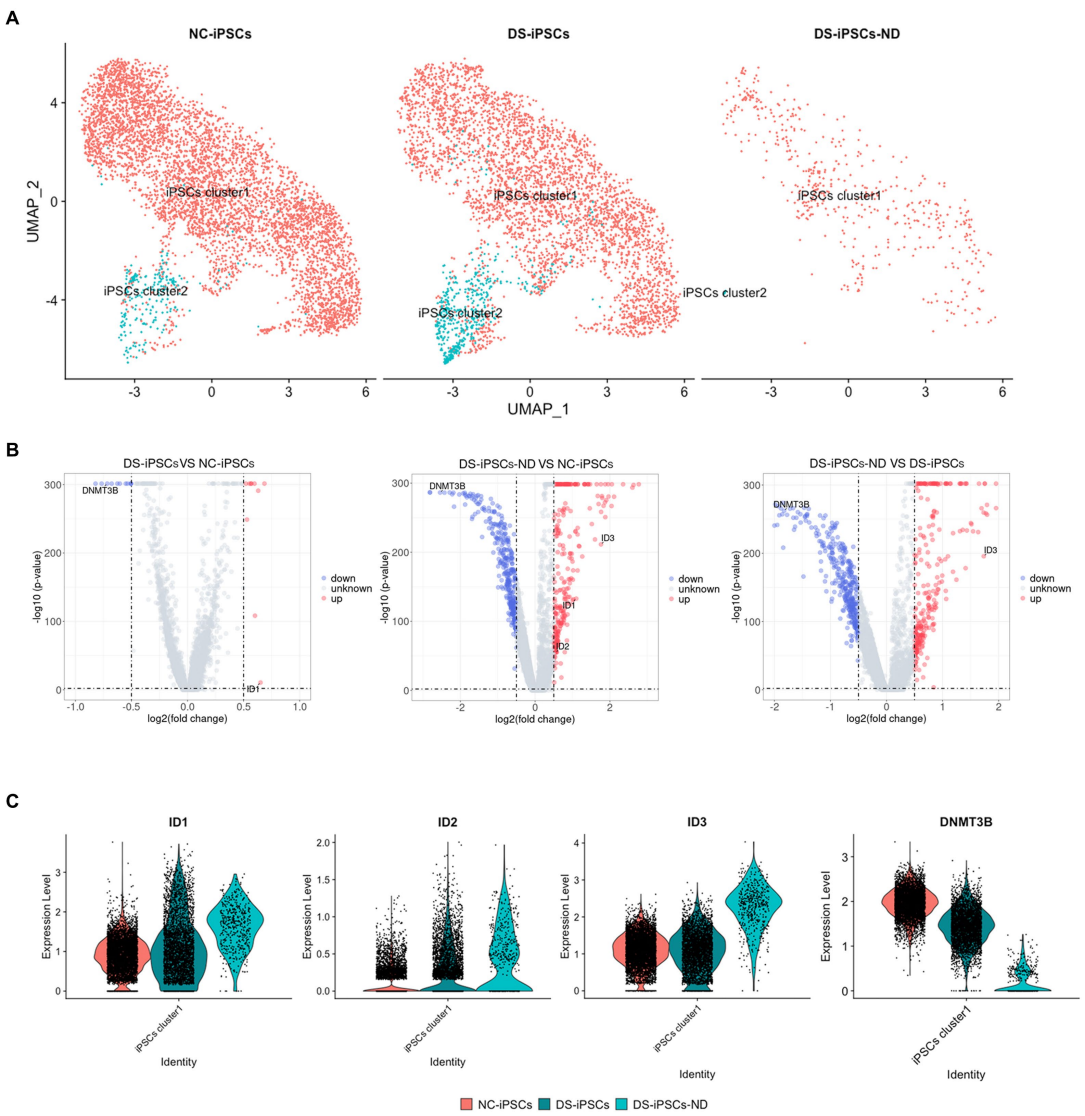
DEG analysis among DS-iPSCs-ND, DS-iPSCs, and NC-iPSCs. **(A)** Mapping DS-iPSCs-ND on iPSC data and identification of the subpopulation. **(B)** DEG analysis among different scenarios on iPSCs cluster1 data, including DS-iPSCs vs. NC-iPSCs, DS-iPSCs-ND vs. NC-iPSCs, and DS-iPSCs-ND vs. DS-iPSCs. **(C)** Expression levels of *DNMT3B*, *ID1*, *ID2*, and *ID3* in DS-iPSCs-ND, DS-iPSCs, and NC-iPSCs on iPSCs cluster1 data. DEG, differentially expressed gene; DS, Down syndrome; iPSC, induced pluripotent stem cell; ND, not differentiated; NC, normal control; DNMT3B, DNA methyltransferase 3β; and ID, inhibitor of differentiation.

Subsequently, DS-iPSCs-NDs were mapped onto the iPSCs cell clusters to assess where these DS-iPSCs-ND originated from. Both DS-iPSCs and NC-iPSCs could be separated into two sub-clusters, which were named iPSCs cluster1 and iPSCs cluster2. The results demonstrated that the two sub-clusters were slightly different with 29 differentially expressed genes (DEGs; Supplementary Table S2). Most DS-iPSCs-ND were identified to have a similar expression profile compared with iPSCs cluster1 (94%; Figure 4A). To investigate the potential underlying mechanism for the repressed differentiation of DS-iPSCs-ND, DEG analysis was further performed among DS-iPSCs-ND, DS-iPSCs cluster1, and NC-iPSCs cluster1.

### DS-iPSCs-ND and DS-iPSCs have abnormal expression patterns compared with NC-iPSCs

The DEG analysis of iPSC cluster1 demonstrated that 21 genes were differentially expressed between the DS-iPSCs and NC-iPSCs, among which 10 genes were overexpressed and 11 genes were under-expressed (Figure 4B; Supplementary Table S3), the expression levels of these differentially expressed genes were also confirmed in another DS-iPSCs line (Supplementary Figure S5). Moreover, 637 genes were differentially expressed between the DS-iPSCs-ND and NC-iPSCs (276 genes were overexpressed and 361 genes were under-expressed; Figure 4B; Supplementary Table S4). Regarding the comparison between DS-iPSCs-ND and DS-iPSCs, there were 280 overexpressed genes and 355 under-expressed genes (Figure 4B; Supplementary Table S5). These results suggested that abnormal expression patterns potentially existed in DS-iPSCs.

DNA methyltransferase 3β (DNMT3B) is thought to function in *de novo* methylation and was demonstrated to be significantly under-expressed in both DS-iPSCs-ND and DS-iPSCs based on the DEG analysis (DS-iPSCs vs. NC-iPSCs, adjusted *p* < 0.001; DS-iPSCs-ND vs. DS-iPSCs: adjusted *p* < 0.001; Figure 4C). Moreover, the trajectory analysis demonstrated that the expression level of *DNMT3B* continuously decreased throughout the whole differentiation process. These results indicated that there may be hypomethylation in DS-iPSCs and their differentiation to NSCs as a result of the under-expression pattern of DNMT3B.

Besides *DNMT3B*, three ID family members: *ID1*, *ID2*, and *ID3* were also identified using DEG analysis (Figure 4C). *ID1* and *ID2* were overexpressed in both DS-iPSCs-ND and DS-iPSCs compared with NC-iPSCs (DS-iPSCs-ND vs. NC-iPSCs, adjusted *p* < 0.001, for *ID1* and *ID2*; DS-iPSCs vs. NC-iPSCs, adjusted *p* = 2.5 × 10^−11^, for *ID1*; Figure 4C). *ID3* exhibited a higher expression level in DS-iPSCs-ND compared with NC-iPSCs, DS-iPSCs and all the other DS-NSC subpopulations (adjusted *p* < 0.001; Figure 4C; Supplementary Figure S6). Trajectory analysis demonstrated that in NC conditions the expression levels of *ID1* and *ID3* gradually decreased during the differentiation process from iPSCs to NSCs. However, in DS conditions, *ID1* and *ID3* exhibited abnormal expression patterns. The expression level of *ID1* decreased at the beginning but then increased for the remainder of the differentiation process. Moreover, for *ID3*, instead of declining, the expression level increased during the whole differentiation process. These results suggested that the over-expression of ID family members, as the negative regulation factor in differentiation, may potentially contribute toward the pathogenesis that impedes differentiation from DS-iPSCs-ND to NSCs.

### Genes related to axon development were differentially expressed in DS-NSCs and DS-RGPCs

**Figure 5:**
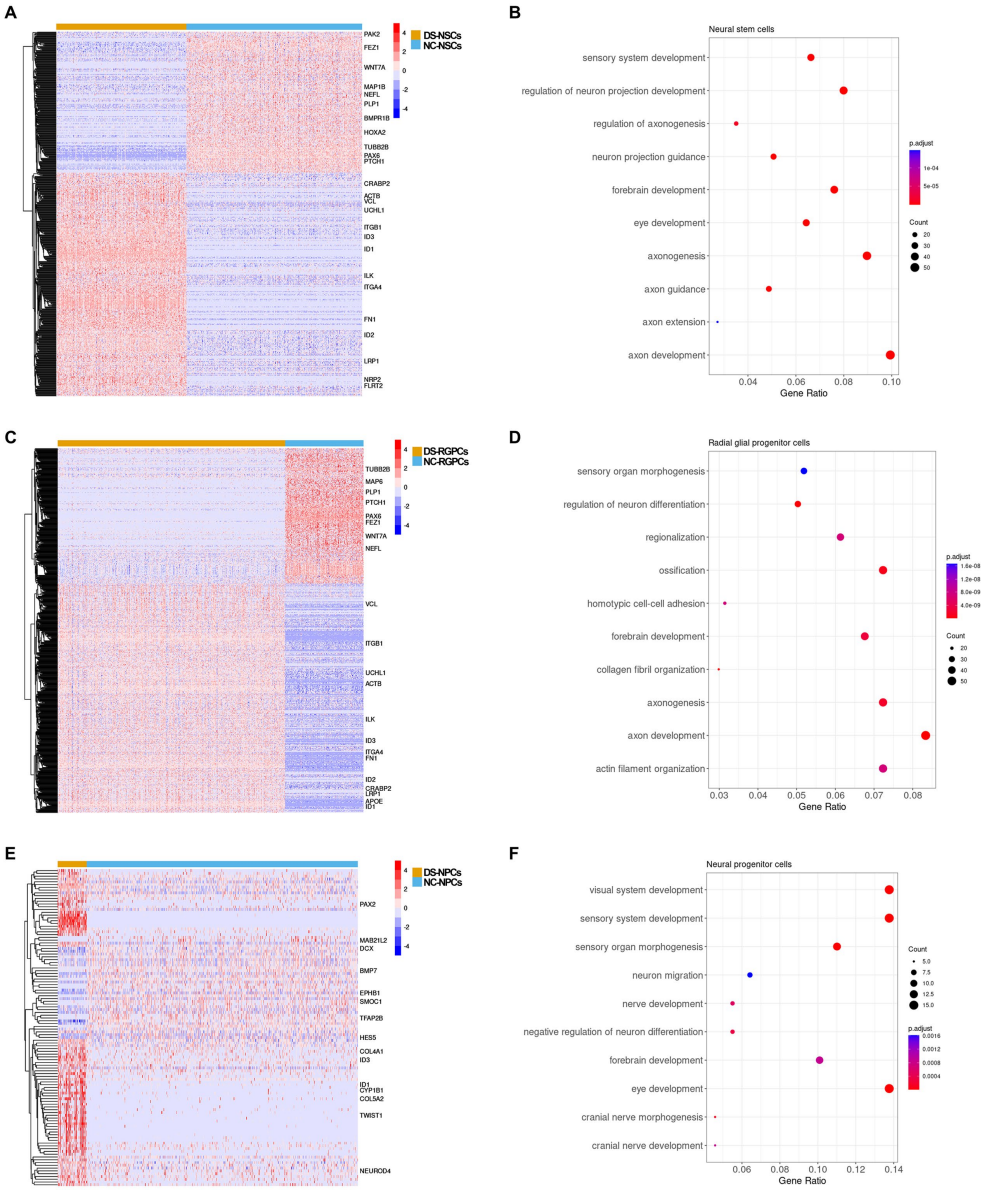
DEG analysis between the subpopulations in DS-NSCs and NC-NSCs. **(A)** Heatmap plot and **(B)** GO enrichment results for DEGs between DS-NSCs and NC-NSCs. **(C)** Heatmap plot and **(D)** GO enrichment result for DEGs between DS-RGPCs and NC-RGPCs. **(E)** Heatmap plot and **(F)** GO enrichment result for DEGs between DS-NPCs and NC-NPCs. DEG, differentially expressed gene; DS, Down syndrome; NSC, neurol stem cell; NC, normal control; GO, gene ontology; RGPC, radial glial progenitor cell; and NPC, neurol progenitor cell.

Subsequently, the gene expression profiles were compared and DEGs were identified between DS and NC cell subpopulations. In total, 561 genes were differentially expressed in DS-NSCs, among which 344 were overexpressed and 217 were under-expressed (Figure 5A). For RGPCs, 442 genes were overexpressed and 260 genes were under-expressed (Figure 5C). GO annotation enrichment analysis demonstrated that DEGs in DS-NSCs and RGPCs were enriched for functions in axon development (Figures 5B,D), such as GO:0061564 “axon development” (DS-NSCs, adjusted *p* = 1.3 × 10^−13^; DS-RGPCs, adjusted *p* = 3.0 × 10^−11^), GO:0007409 “axonogenesis” (DS-NSCs adjusted *p* = 1.9 × 10^−12^; DS-RGPCs, adjusted *p* = 1.7 × 10^−9^), and GO:0048675 “axon extension” (DS-NSCs, adjusted *p* = 1.5 × 10^−4^). Genes that contribute to axonal pathfinding and normal axonal guidance were under-expressed in DS-NSCs and DS-RGPCs, including *PAX6*, which is required for the development of axonal connections (Jones et al., 2002), microtubule-associated protein 6, which was previously reported in axons and shown to stabilize microtubules (Hamdan et al., 2020) and *WNT7A*, which serves an important role in axon development, guidance, and axonal remodeling (Qu et al., 2013). However, those genes that would have a negative function in nervous system development were overexpressed in DS-NSCs and DS-RGPCs, such as *ID1*, *ID2*, and *ID3*.

### DS-NPCs were dysfunctional in visual system development

There were 114 DEGs identified between the DS-NPCs and NC-NPCs, among which 80 genes were overexpressed and 34 genes were under-expressed (Figure 5E). The results of the functional analysis demonstrated that the DEGs in the DS-NPCs were related to the development of the visual system (Figure 5F), such as GO:0150063 “visual system development” (adjusted *p* = 2.3 × 10^−6^), GO:0001654 “eye development” (adjusted p = 2.3 × 10^−6^), and GO:0090596 “sensory organ morphogenesis” (adjusted *p* = 1.1 × 10^−5^). DEGs in DS-NPCs were also demonstrated to be involved in the development of the cranial nerve (adjusted *p* = 8.7 × 10^−4^) where the optic nerve belongs to.

Those genes which served an essential role in eye development were under-expressed in DS-NPCs, including bone morphogenetic protein 7 (*BMP7*) (Wyatt et al., 2010) and *DCX* (Sanchez-Farias and Candal, 2015). Moreover, *ID1* and *ID3* were demonstrated to be overexpressed in DS-NPCs.

### ID family members might prevent DS-iPSCs from differentiating into NSCs

DS-iPSCs-ND were a cluster of DS-iPSCs that were not able to differentiate into DS-NSCs. Compared with NC-iPSCs, three ID family members were demonstrated to be overexpressed in DS-iPSCs-ND. Furthermore, they had abnormal expression patterns during the whole differentiation process from iPSCs to NSCs. Therefore, as a negative regulation factor, the overexpression of ID family members may prevent DS-iPSCs-ND from differentiating into NSCs.

The ID family members were also demonstrated to be overexpressed in the DS-NSCs dataset. *ID1* was overexpressed in DS-NSCs, DS-NPCs, and DS-RGPCs. *ID2* was overexpressed in DS-NSCs and DS-RGPCs and *ID3* had a higher expression level in almost all subpopulations of DS-NSCs, including DS-NSCs, DS-NPCs, DS-RGPCs, and DS-iPSCs-ND. These results suggested that *ID1*, *ID2*, and *ID3* potentially served important functions in DS.

Then, we tried to validate our results by an independent dataset. We found that ID1 and ID3 gene was significantly differentially expressed in endothelial cells of DS aging brain. ID2 gene was significantly differentially expressed in astrocytes of DS aging brain (Supplementary Figure S7; Palmer et al., 2021). These results again proved that ID family genes play an important role in DS pathology.

To further confirm the abnormal expression of ID family members, RT-qPCR and Western blot were performed. The results demonstrated that *ID1*, *ID2*, and *ID3* were all significantly overexpressed in both DS-iPSCs and DS-NSCs (Supplementary Figure S8) and ID1 was overexpressed in two independent DS-iPSCs lines (Supplementary Figure S9), which to an extent supported the hypothesis.

## Discussion

The molecular mechanisms behind the neurodevelopmental disorder of DS are still unclear. Due to the lack of suitable technology, previous studies concerning neural differentiation in DS were only performed at the cell-population level and therefore the information about the diversity, heterogeneity and dynamics of single cells was lost. However, it has been confirmed that, during the neural differentiation process, cells contain different subpopulations that have the potential for multipotency of differentiation into neurons, oligodendrocytes and astrocytes. Therefore, in the present study, scRNA-seq was applied to NSCs, which were differentiated from the iPSCs of a patient with DS and a normal control, and the disorder and molecular mechanisms of DS were analyzed at the single-cell level. The properties in DS-NSCs were investigated and the abnormalities in the main cell subpopulations throughout the differential process from DS-iPSCs to DS-NSCs, such as DS-NPCs, DS-RGPCs, and DS-iPSC-ND were explored.

### Abnormal differentiation fate of DS-NSCs causes the overrepresentation of glial lineages and impaired neurogenesis in DS

The present study demonstrated there was abnormal neural differentiation fate in DS, which led to the under-representation of DS-NPCs in the patient with DS. It has previously been well studied that the brain of patients with DS is characterized by the hypotrophy and hypocellularity of neurons (Stagni et al., 2018; Baburamani et al., 2019). A substantial increase in the number of glial fibrillary acid protein (GFAP) positive cells, both astrocytes and radial glial cells, was found in fetuses with DS at 18–20 weeks of gestation and all age range samples of DS brains (Mito and Becker, 1993; Dossi et al., 2018; Ponroy Bally et al., 2020). This indicates that the imbalance between neurons and glial cells is an early event during fetal development. Based on the identification of cell subpopulations in the present study, DS-NSCs were demonstrated to have a large fraction of RGPCs (38.1%), which can produce certain lineages of glia, including astrocytes and oligodendrocytes. However, in NC-NSCs, there were only 13.9% RGPCs. However, NC-NSCs had 19.5% NPCs, but DS-NSCs only consisted of 1.9% NPCs.

While the occurrence of a gliogenic shift in the neural population derived from DS-iPSCs has been reported in several studies, the specific populations and timing of appearance of glial cells is uncertianed (Briggs et al., 2013; Weick et al., 2013; Hibaoui et al., 2014). In recent research, Mollo et al. (2021) reported the over-representation of glial cells at 21 days from the onset of iPSC neural induction based on the overexpression of the glial markers S100 calcium binding protein B, oligodendrocyte transcription factor (*OLIG*)*1*, *OLIG2*, *GFAP*, *CNPase*, and platelet-derived growth factor receptor α. In the present study, using scRNA-seq technology, it was reported that the gliogenic shift in the neural population occurred 7 days following the onset of iPSC neural induction, which was much earlier than what was expected based on the aforementioned previous studies. Since iPSCs behave like embryonic cells (ECs), the imbalance between neurons and glial cells in the brains of patients with DS may potentially be caused by the abnormal differentiation of ECs to NSCs during embryo development.

### ID1, ID2, and ID3 interrupt the differentiation of DS-iPSCs to DS-NSCs

Based on scRNA-seq in the present study, it was observed that a cluster of DS-iPSCs were repressed during the process of differentiation into DS-NSCs and were named DS-iPSCs-ND. With further DEG analysis, the ID family members, *ID1*, *ID2* and *ID3* were demonstrated to be significantly overexpressed in both DS-iPSCs-ND and all the other subpopulations of DS-NSCs. The overexpression of the ID family could potentially be the reason for the repressed differentiation of DS-iPSCs-ND and the gliogenic shift in the neural population in the brains of DS patients.

ID genes, which include four members (*ID1*-*ID4*) in mammalian cells, are necessary for the regulation of differentiation during embryogenesis (Perk et al., 2005). The expression patterns of *ID1*, *ID2*, and *ID3* are significantly different from that of *ID4* during embryogenesis (Jen et al., 1996), which indicates that *ID1*, *ID2*, and *ID3* may have a similar function. *ID1*, *ID2*, and *ID3* serve roles in the development of the nervous system (Lyden et al., 1999; Nam and Benezra, 2009; Chen et al., 2012; Bohrer et al., 2015).

A gradually decreasing gradient of *ID1* levels along the neurogenic lineages was reported previously (Nam and Benezra, 2009). This corresponds to the results in the present study, whereby the expression levels of *ID1* decreased during differentiation from NC-iPSCs to NC-NSCs. Therefore, the overexpression of *ID1* in DS-iPSCs-ND may harm the differentiation of neuron cells. In Alzheimer’s disease (AD) the overexpression of *ID1* triggers the apoptosis of neuron cells by activating hypoxia-inducible factor-1α and increasing the expression of the sonic hedgehog protein (Chen et al., 2020).

In the present study, the overexpression of *ID3* was observed in DS-iPSCs and DS-NSCs and it may therefore promote DS-NESC differentiation into astrocytes according to a previous study (Bohrer et al., 2015). Therefore, the overexpression of *ID3* may lead to an increase in the number of astrocytes and result in a gliogenic shift in the neural population in the brains of patients with DS (Mito and Becker, 1993).

### Disorder of DS-NPCs causes abnormalities of the optic nerve in patients with DS

As well as the disorders identified during the whole neural differentiation process, the abnormality of DS in each subpopulation of NSCs was also analyzed. Among them, the NPCs were demonstrated to be an important subpopulation, whereby they are the source of differentiation potential to neurons. In the present study, it was demonstrated that DEGs in DS-NPCs were enriched for eye and visual system development with differential expression analysis. Those genes which served an essential role in eye development were under-expressed in DS-NPCs, including *BMP7* and *DCX*.

Abnormalities of the optic nerve in children with DS have previously been reported. Visual acuity is significantly lower in children with DS compared with controls and visual acuity is diminished in 80% of children with DS (Postolache, 2019). Moreover, adults with DS also have variously severe vision deficits, including reduced sensitivity across spatial frequencies and temporal modulation rates, reduced stereopsis, impaired vernier acuity, and anomalies in color discrimination (Krinsky-McHale et al., 2014).

The pattern of vision deficits seen in adults with DS was also found to be similar to that in adults with AD without intellectual disability (Rocco et al., 1997). This similarity has been attributed to the presence of the neuropathology of AD, which is observed in adults with DS as early as their fifth decade of life (Krinsky-McHale et al., 2014).

In the present study, it was demonstrated that the abnormalities of the optic nerve in patients with DS may be caused by differentially expressed visual development-related genes in DS-NPCs. Besides being responsible for neurogenesis in the visual cortex of the brain, NPCs are also able to differentiate into specific retinal neurons and their differentiation along retinal sub-lineages is significantly influenced when co-cultured with embryonic or neonatal retinal cells (Ahmad et al., 2004). Therefore, the under-expressed visual development-related genes may cause the abnormal differentiation of DS-NPCs to visual cortex neurons and specific retinal neurons, which would lead to the dysfunction of the optic nerve in patients with DS. It was also demonstrated in the present study that DS-NPCs have a disorder in cranial nerve development. Since the optic nerve is the second pair of 12 cranial nerve pairs, a disorder in cranial nerve development would also lead to abnormalities of the optic nerve in patients with DS (Herrera et al., 2019). The results of the present study concerning the differentially expressed visual development-related genes in DS-NPCs, provided a possible explanation for the underlying mechanism behind the abnormalities of the optic nerve in patients with DS.

ln conclusion, the present study, using scRNA-seq, analyzed the disorder of DS at a single-cell resolution level. As well as the abnormalities reported in DS-NSCs, the present study demonstrated that a cluster of DS-iPSCs (DS-iPSCs-ND) were not able to differentiate into DS-NSCs. With further DEG analysis, ID family members were demonstrated to be significantly overexpressed in both DS-iPSCs-ND and all other subpopulations of DS-NSCs. These cells exhibited an abnormal expression pattern during the entire differentiation process from DS-iPSCs into DS-NSCs. These results indicated that ID family members may be the key factor that interrupts neural differentiation in DS, which results in the repressed differentiation of DS-iPSCs-ND and the gliogenic shift in the neural population. The present study provided novel information and therefore contributed to the understanding of DS and other related neurodevelopmental disorders.

## Data availability statement

The data that support the findings of this study are openly available in Gene Expression Omnibus at https://www.ncbi.nlm.nih.gov/geo/query/acc.cgi?acc=GSE208625, reference number GSE208625.

## Author contributions

J-bY and FZ supervised the project and revised the manuscript. J-jQ conducted the research, analyzed the data, and wrote the manuscript. Y-nL did the validation experiment. HW provided some useful suggestions and ideas on the data analysis. All authors contributed to the article and approved the submitted version.

## Funding

J-bY is supported by the grant from the National Natural Science Foundation of China (81971421 and 81471485). FZ is supported by the grants from the National Key Research and Development Program of China (2019YFA0801402), Innovative Research Team of High-Level Local Universities in Shanghai (SHSMU-ZDCX20212200), and Shanghai key clinical specialty project (shslczdzk05705). J-jQ is sponsored by Shanghai Sailing Program (21YF1424200). The funders had no role in study design, data collection and analysis, decision to publish, or preparation of the manuscript.

## Conflict of interest

The authors declare that the research was conducted in the absence of any commercial or financial relationships that could be construed as a potential conflict of interest.

## Publisher’s note

All claims expressed in this article are solely those of the authors and do not necessarily represent those of their affiliated organizations, or those of the publisher, the editors and the reviewers. Any product that may be evaluated in this article, or claim that may be made by its manufacturer, is not guaranteed or endorsed by the publisher.
