# Supplementary figures and images for: Single-cell RNA sequencing of neural stem cells derived from human trisomic iPSCs reveals the abnormalities during neural differentiation of Down syndrome
Source: Front Mol Neurosci. 2023 Jun 15;16:1137123. doi: 10.3389/fnmol.2023.1137123 (PMC10311021; doi:10.3389/fnmol.2023.1137123)

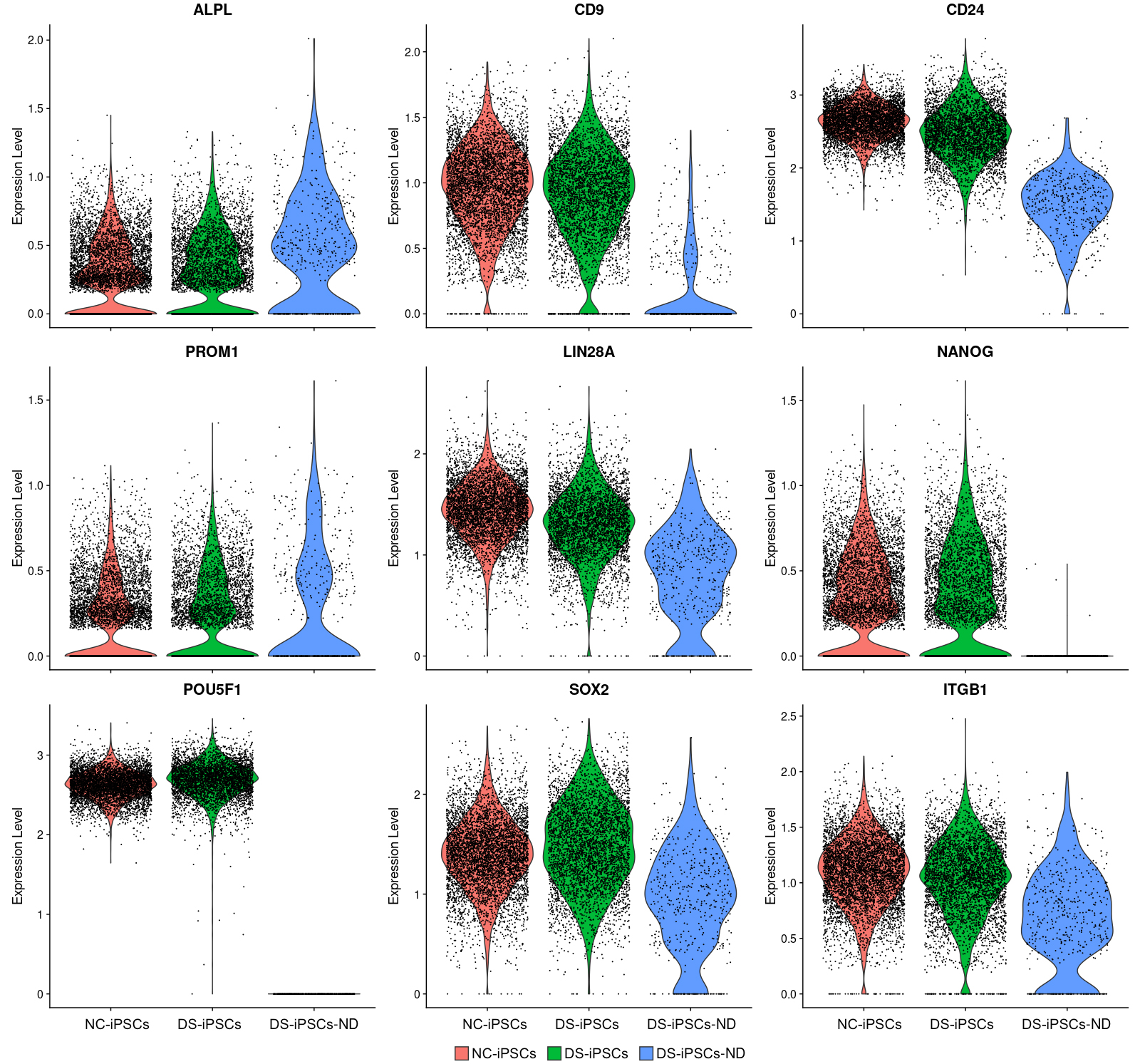

Supplement: Supplementary file 1 [file Image_1.JPEG]

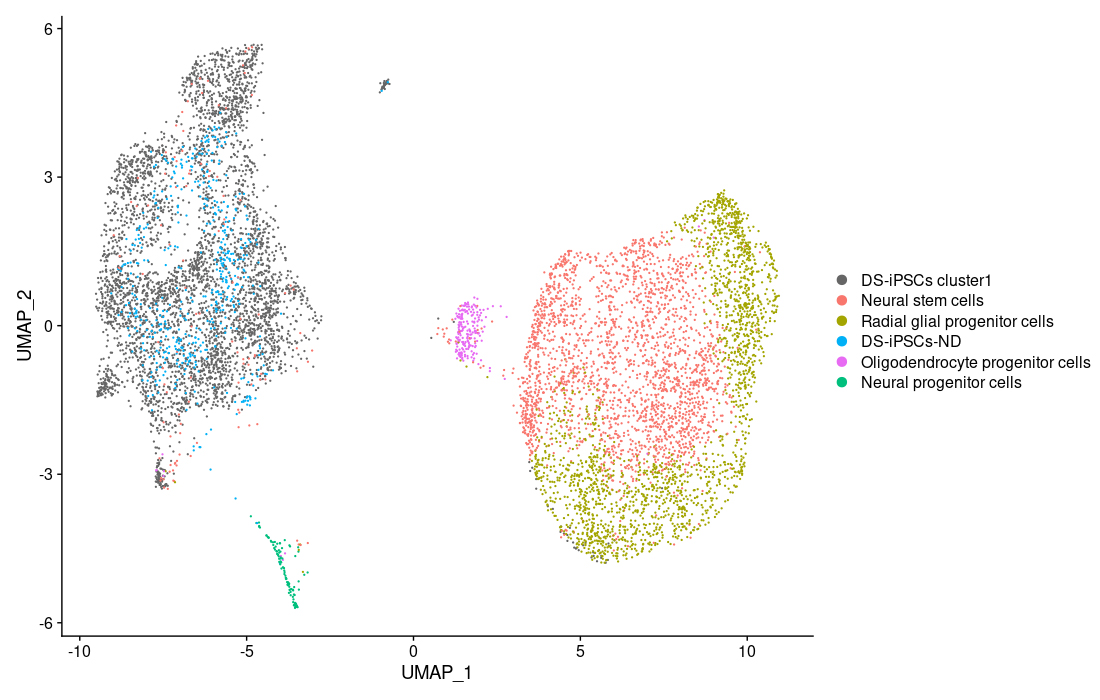

Supplement: Supplementary file 2 [file Image_2.JPEG]

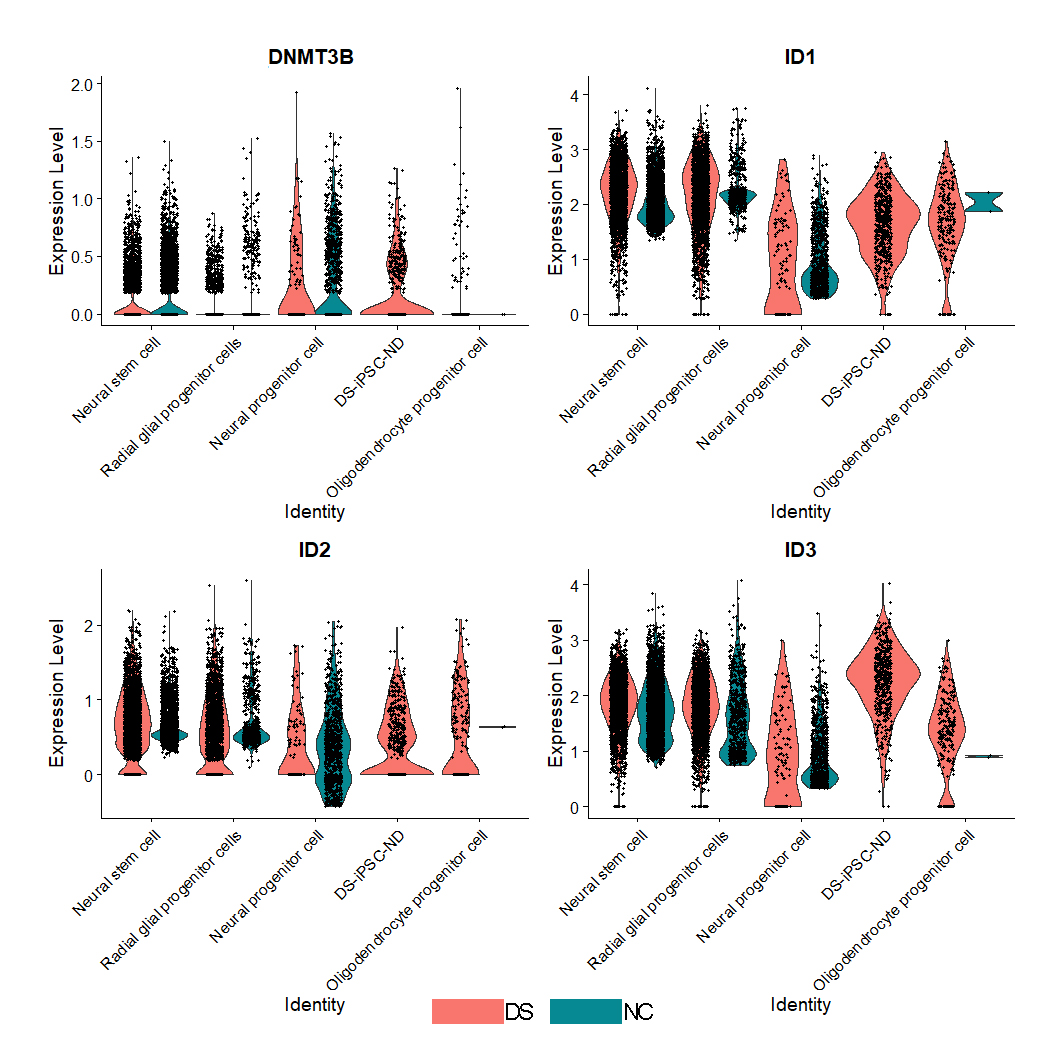

Supplement: Supplementary file 3 [file Image_3.JPEG]

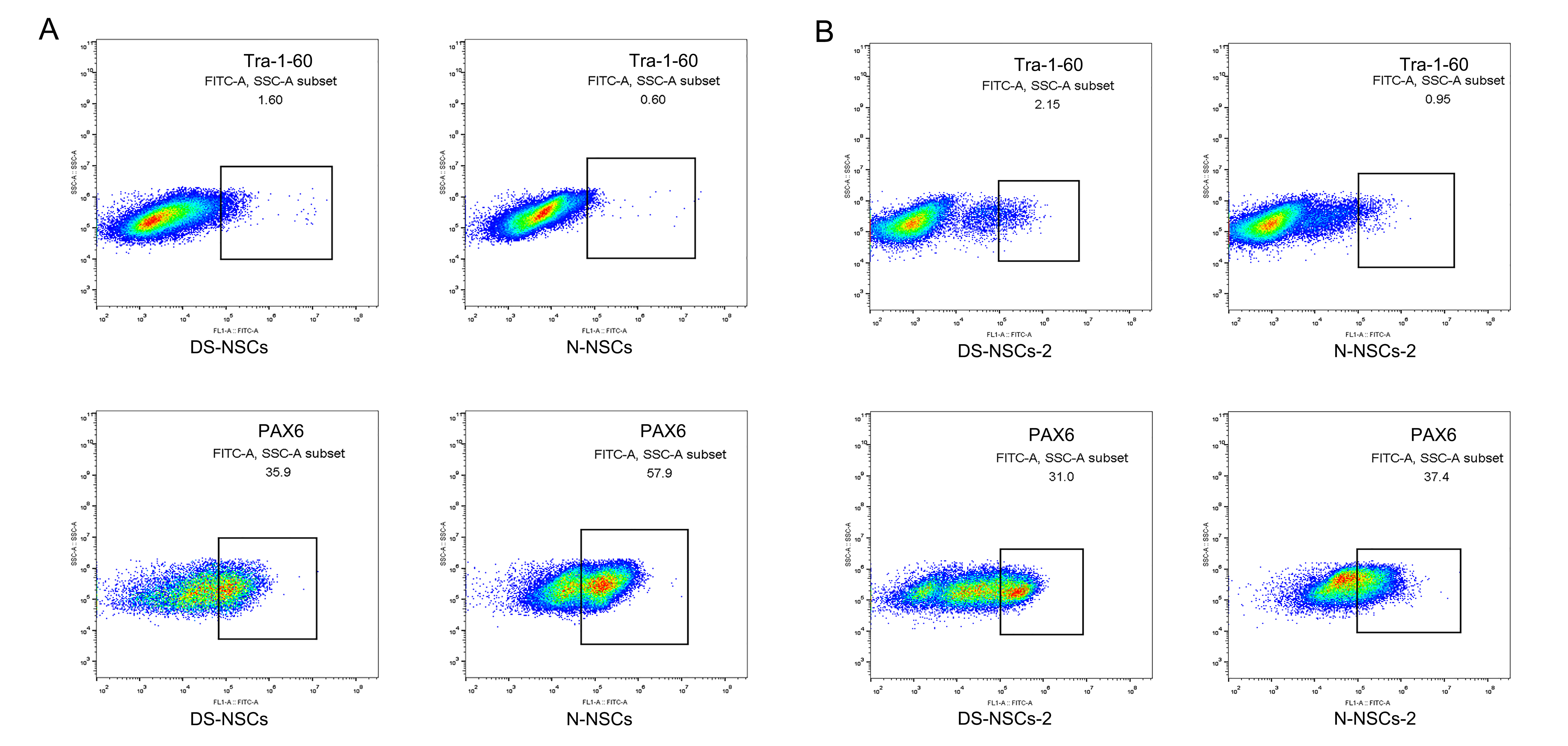

Supplement: Supplementary file 4 [file Image_4.JPEG]

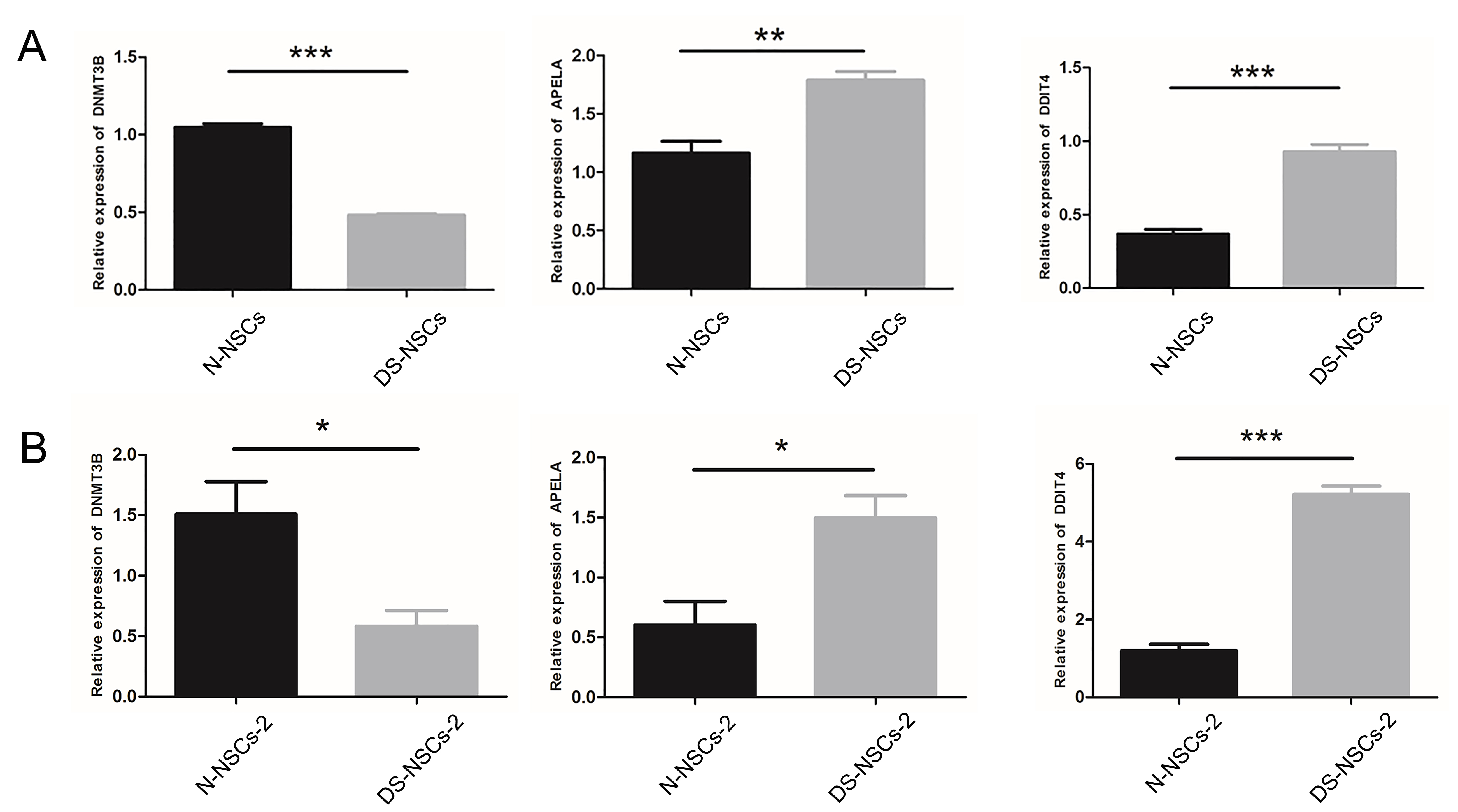

Supplement: Supplementary file 5 [file Image_5.JPEG]

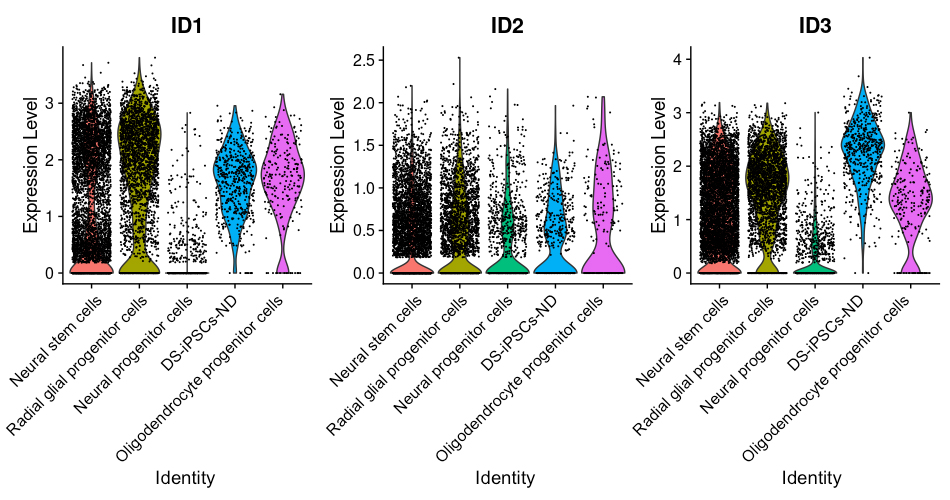

Supplement: Supplementary file 6 [file Image_6.JPEG]

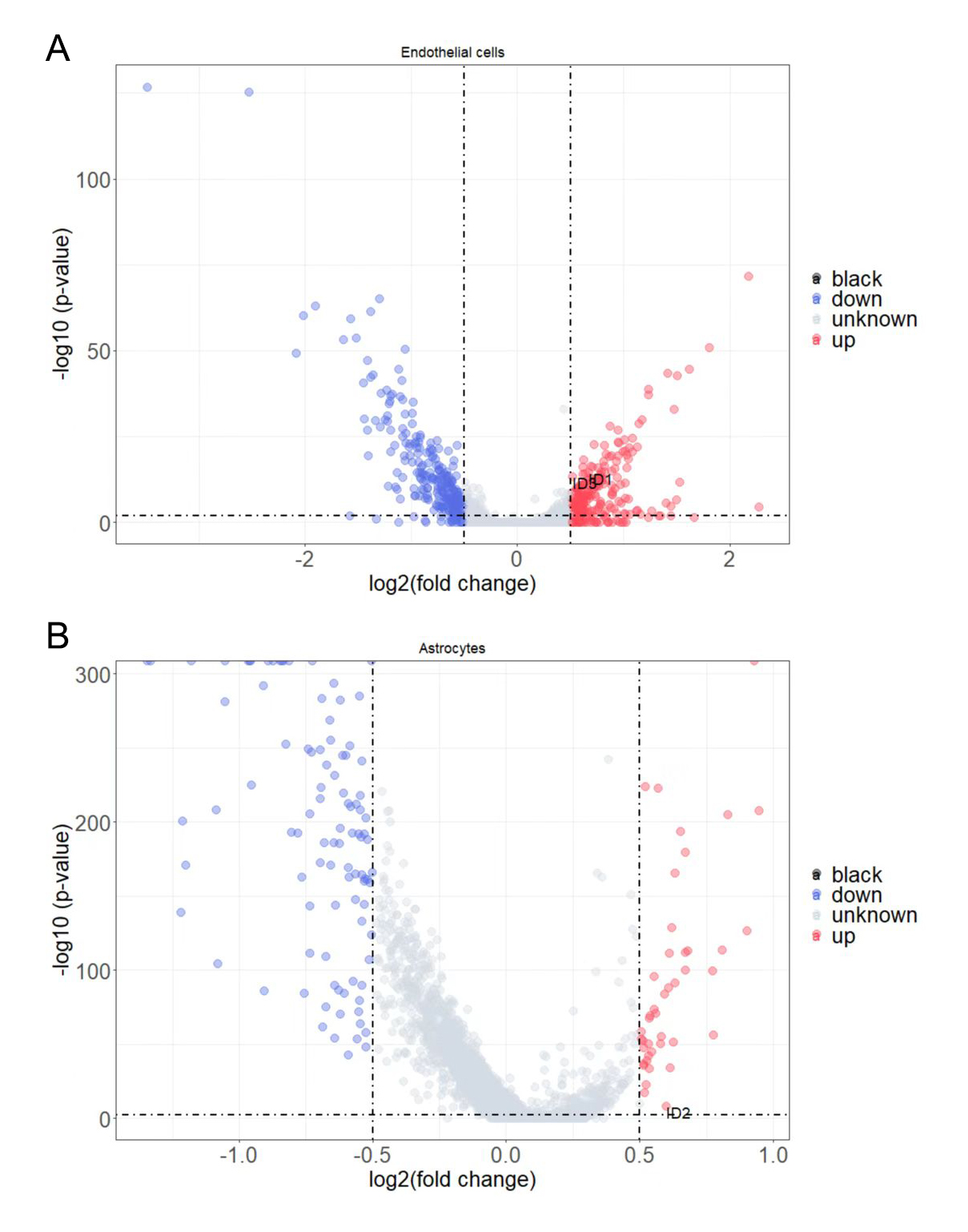

Supplement: Supplementary file 7 [file Image_7.JPEG]

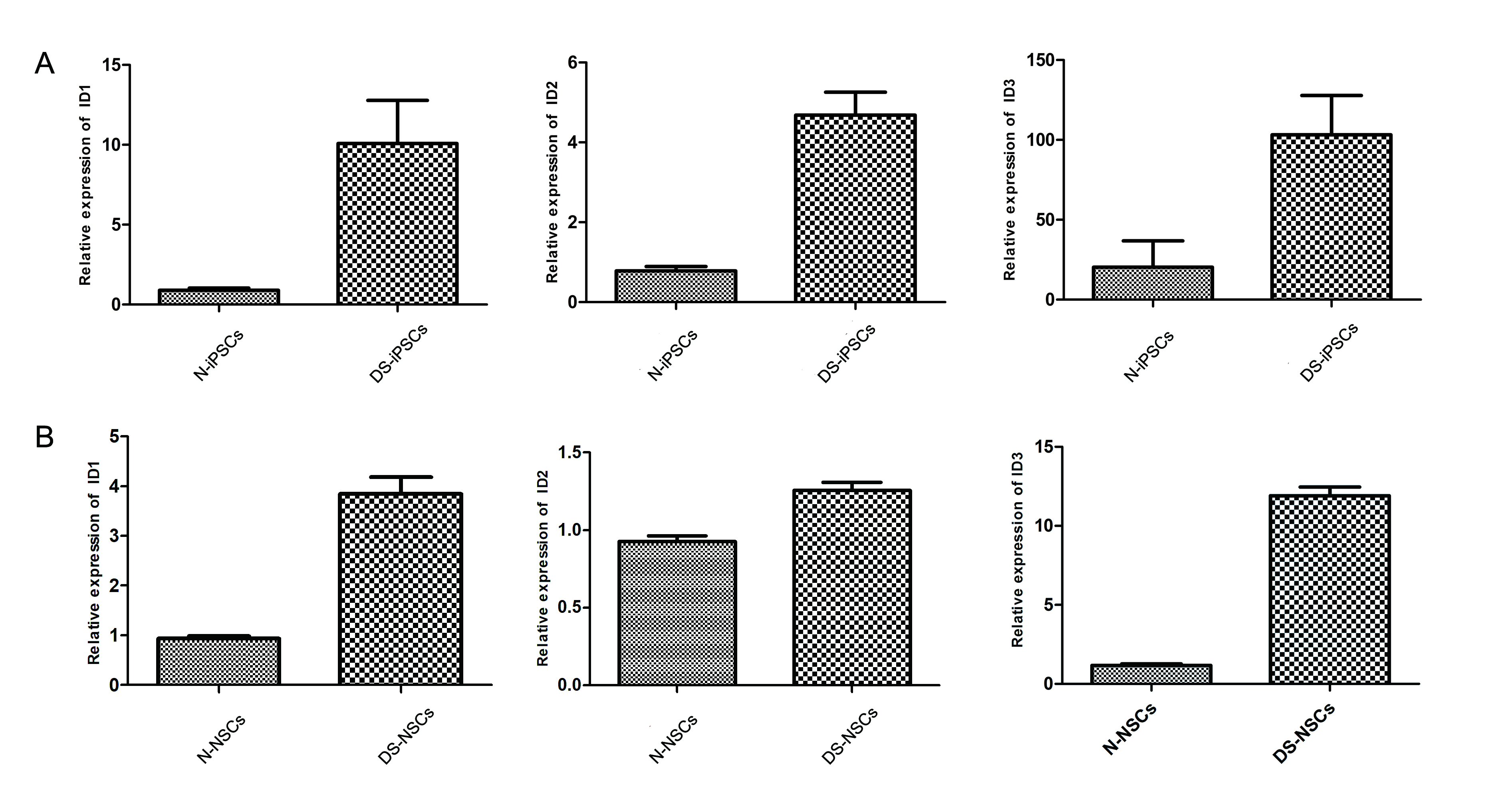

Supplement: Supplementary file 8 [file Image_8.JPEG]

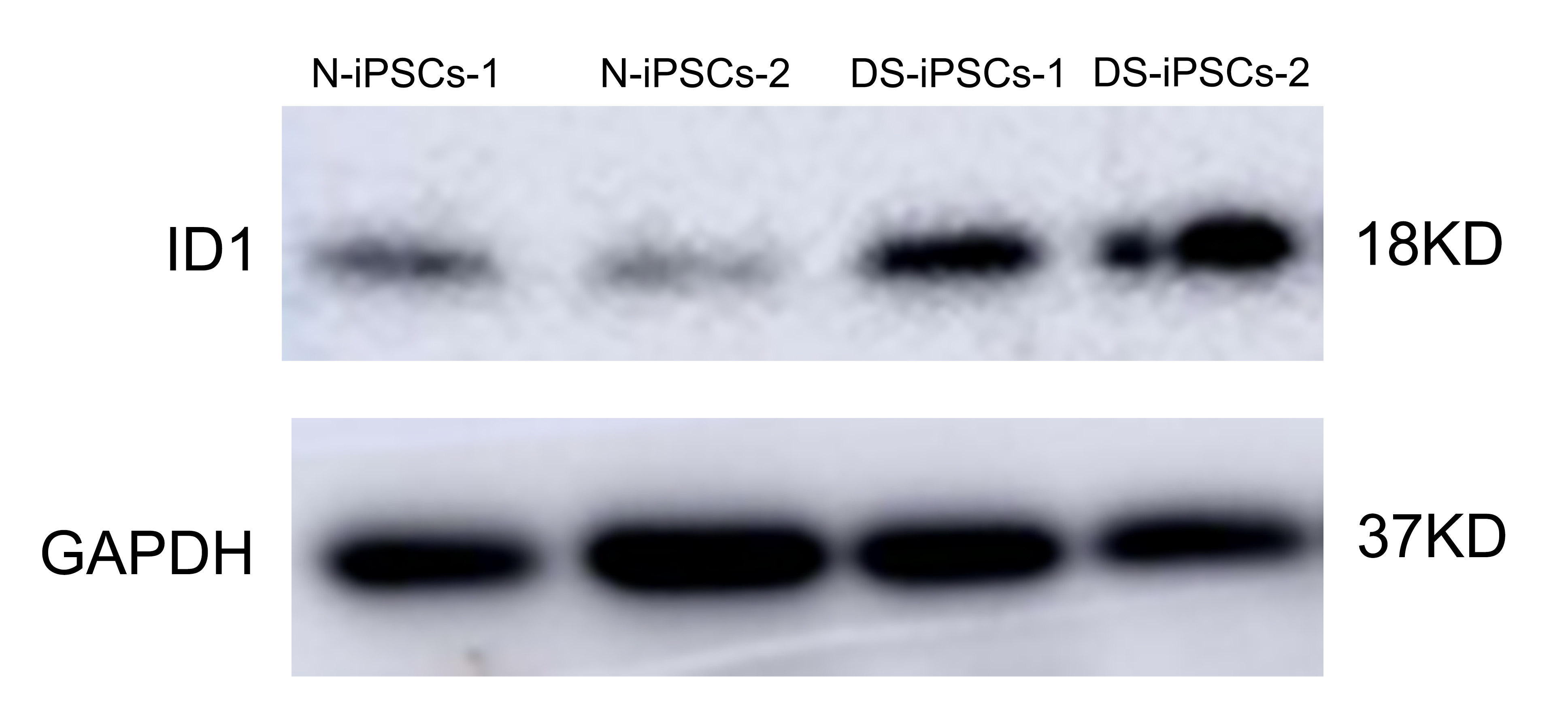

Supplement: Supplementary file 9 [file Image_9.JPEG]
